# Radiomic study of antenatal prediction of severe placenta accreta spectrum from MRI

**DOI:** 10.1093/bjr/tqae164

**Published:** 2024-08-17

**Authors:** Helena C Bartels, Eric Wolsztynski, Jim O’Doherty, David P Brophy, Roisin MacDermott, David Atallah, Souha Saliba, Nadine El Kassis, Malak Moubarak, Constance Young, Paul Downey, Jennifer Donnelly, Tony Geoghegan, Donal J Brennan, Kathleen M Curran

**Affiliations:** Department of UCD Obstetrics and Gynaecology, School of Medicine, University College Dublin, National Maternity Hospital, Dublin 2, Ireland; School of Mathematical Sciences, University College Cork, Cork T12 XF62, Ireland; Insight SFI Centre for Data Analytics, Dublin, Ireland; Siemens Medical Solutions, Malvern, PA 19355, United States; Department of Radiology & Radiological Science, Medical University of South Carolina, Charleston, SC 29425, United States; Radiography & Diagnostic Imaging, University College Dublin, Dublin D04 V1W8, Ireland; Department of Radiology, St Vincents University Hospital, Dublin D04 T6F4, Ireland; Department of Radiology, St Vincents University Hospital, Dublin D04 T6F4, Ireland; Department of Gynecology and Obstetrics, Hôtel-Dieu de France University Hospital, Saint Joseph University, Beirut, Lebanon; Department of Radiology: Fetal and Placental Imaging, Hôtel-Dieu de France University Hospital, Saint Joseph University, Beirut, Lebanon; Department of Gynecology and Obstetrics, Hôtel-Dieu de France University Hospital, Saint Joseph University, Beirut, Lebanon; Department of Gynecology and Obstetrics, Hôtel-Dieu de France University Hospital, Saint Joseph University, Beirut, Lebanon; Kliniken Essen Mitte, Department of Gynecology and Gynecologic Oncology, Essen, Germany; Department of Histopathology, National Maternity Hospital, Dublin D02 YH21, Ireland; Department of Histopathology, National Maternity Hospital, Dublin D02 YH21, Ireland; Department of Obstetrics and Gynaecology, Rotunda Hospital, Dublin D01 P5W9, Ireland; Department of Radiology, Mater Misericordiae University Hospital, Dublin D07 AX57, Ireland; Department of UCD Obstetrics and Gynaecology, School of Medicine, University College Dublin, National Maternity Hospital, Dublin 2, Ireland; University College Dublin Gynaecological Oncology Group (UCD-GOG), Mater Misericordiae University Hospital and St Vincent’s University Hospital, Dublin, Ireland; Systems Biology Ireland, School of Medicine, University College Dublin, Dublin D04 V1W8, Ireland; School of Medicine, University College Dublin, Dublin D04 V1W8, Ireland

**Keywords:** placenta accreta spectrum, radiomics, machine learning, MRI, pregnancy

## Abstract

**Objectives:**

We previously demonstrated the potential of radiomics for the prediction of severe histological placenta accreta spectrum (PAS) subtypes using T2-weighted MRI. We aim to validate our model using an additional dataset. Secondly, we explore whether the performance is improved using a new approach to develop a new multivariate radiomics model.

**Methods:**

Multi-centre retrospective analysis was conducted between 2018 and 2023. Inclusion criteria: MRI performed for suspicion of PAS from ultrasound, clinical findings of PAS at laparotomy and/or histopathological confirmation. Radiomic features were extracted from T2-weighted MRI. The previous multivariate model was validated. Secondly, a 5-radiomic feature random forest classifier was selected from a randomized feature selection scheme to predict invasive placenta increta PAS cases. Prediction performance was assessed based on several metrics including area under the curve (AUC) of the receiver operating characteristic curve (ROC), sensitivity, and specificity.

**Results:**

We present 100 women [mean age 34.6 (±3.9) with PAS], 64 of whom had placenta increta. Firstly, we validated the previous multivariate model and found that a support vector machine classifier had a sensitivity of 0.620 (95% CI: 0.068; 1.0), specificity of 0.619 (95% CI: 0.059; 1.0), an AUC of 0.671 (95% CI: 0.440; 0.922), and accuracy of 0.602 (95% CI: 0.353; 0.817) for predicting placenta increta. From the new multivariate model, the best 5-feature subset was selected *via* the random subset feature selection scheme comprised of 4 radiomic features and 1 clinical variable (number of previous caesareans). This clinical-radiomic model achieved an AUC of 0.713 (95% CI: 0.551; 0.854), accuracy of 0.695 (95% CI 0.563; 0.793), sensitivity of 0.843 (95% CI 0.682; 0.990), and specificity of 0.447 (95% CI 0.167; 0.667).

**Conclusion:**

We validated our previous model and present a new multivariate radiomic model for the prediction of severe placenta increta from a well-defined, cohort of PAS cases.

**Advances in knowledge:**

Radiomic features demonstrate good predictive potential for identifying placenta increta. This suggests radiomics may be a useful adjunct to clinicians caring for women with this high-risk pregnancy condition.

## Introduction

Placenta accreta spectrum (PAS) describes a condition of abnormal placental adherence or invasion into the myometrium.^1^ Cases are classified clinically, at intraoperative assessment, and on histopathology.^2^^,^^3^ Although rare, PAS is a major cause of maternal morbidity, largely as a consequence of major obstetric haemorrhage and surgical morbidity.^4^^,^^5^

Antenatal diagnosis relies heavily on imaging expertise^6^^,^^7^ and up to 50% of cases worldwide remain undiagnosed during pregnancy.^4^ Antenatal diagnosis is one of the key predictors of improved maternal outcomes and therefore efforts to increase diagnosis rates,^4^ as well as identify severe cases antenatally, are important.

The 2 main imaging modalities used to assess PAS during pregnancy are ultrasound and MRI, and well-defined criteria for both have now been described.^6^^,^^7^ As ultrasound is routinely performed in pregnancy for assessing the foetus, many cases of PAS will first be suspected from ultrasound features.^6^^,^^8^ In expert hands, up to 1 in 5 cases of PAS may not be diagnosed from ultrasound.^9^ Furthermore, predicting disease severity and distinguishing cases of PAS from placenta previa with scar dehiscence remains challenging, as these cases will have similar ultrasound findings such as myometrial thinning and loss of the clear zone.^10^ Hence, ultrasound relies heavily on clinician expertise.^11^

The role and value of MRI may vary between institutions, depending on familiarity with ultrasound, equipment, and availability of radiological expertise. However, literature suggests it may have particular value in areas such as delineating posterior or lateral defects and for surgical planning.^7^^,^^12^^,^^13^ As PAS remains a rare disease, some practitioners will see less than 1-2 cases per year.^14^ Therefore, it is not surprizing that, even in specialist centres, MRI may incorrectly classify PAS cases in up to 30% of cases.^15^

Radiomics is an emerging field in image diagnostics, where potential image biomarkers are extracted from medical images.^16^ MRI radiomics studies have shown promising results for diagnosing PAS and predicting clinical outcomes such as hysterectomy and blood loss.^17–20^ A metanalysis summarizing 10 PAS MR radiomics studies highlighted a number of limitations of the existing studies, such as lack of availability of histopathology, which is the gold standard for diagnosing PAS, and variation in the radiomic methodology used.^21^ Our group previously investigated some of these limitations using a well-classified PAS cohort, however given the restrictive sample size, our performance metrics were moderate.^22^

This study aims to consolidate our recent findings on the feasibility of radiomics modelling to predict severe histopathological subtypes of PAS. We use a previously published image segmentation protocol,^22^ international standards for defining PAS,^2^ with radiomic feature extraction performed using standardized methods.^23^ We focus on developing a new multivariate model using random subset feature selection to predict severe placenta increta subtypes PAS cases.

## Methods

### Study population

**Figure 1.**
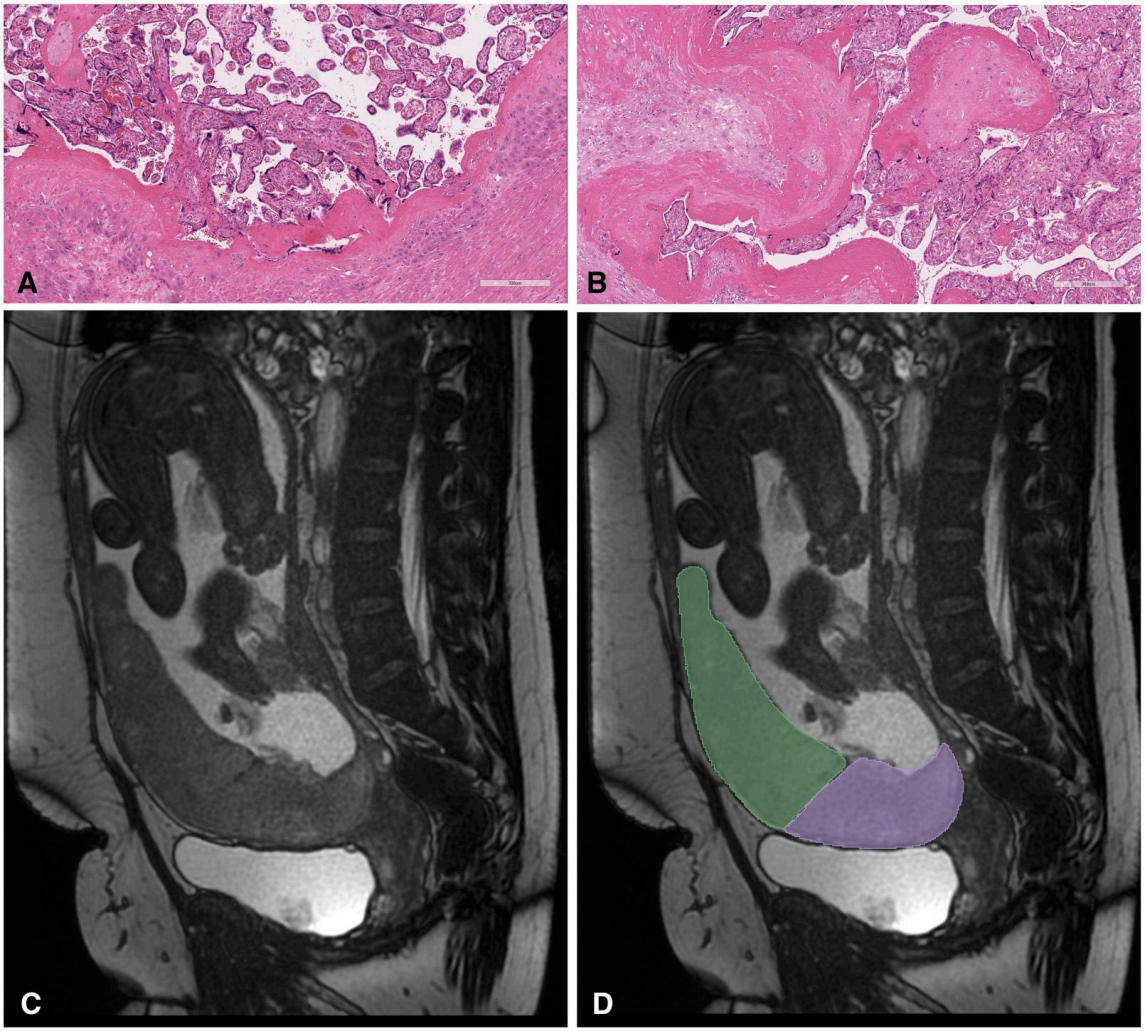
Histopathology of accreta and increta case and MRI segmentations. (A) Haematoxylin and eosin stained slide at high 200 µm power. Placental villi are seen in direct contact with the myometrium with no decidua present in a case of histopathological placenta accreta. (B) Haematoxylin and eosin stained slide at high 200 µm power. Placental villi are seen deep within the myometrium with no intervening decidua in a case of histopathological placenta increta. (C and D) An example of the placental regions of interest from which radiomic features were extracted. (C) Sagittal, b-SSFP sequence, of PAS case at 30 weeks’ gestation. The placenta is anterior completely covering the internal cervical os (placenta previa). (D) Inferior (lower, purple) and superior (upper, green) placental regions of interest. PAS = placenta accreta spectrum.

Ethical approval was obtained from the hospital ethics committees (EC30.2018, RAG.2019-10, CEHDF.2071) and participants provided written informed consent. The study was conducted according to the principles expressed in the Declaration of Helsinki. This is a multi-centre study consisting of 3 separate cohorts, 2 obtained from 2 centres in Ireland [centres 1 & 2 (*n* = 44)] and another dataset obtained from Lebanon [centre 3 (*n* = 56)]. For centres 1 & 2, data were prospectively obtained between January 2018 and August 2023, and 41 cases were included in a previous radiomic analysis.^22^ Inclusion criteria were as follows: women who underwent MRI for suspicion of PAS based on ultrasound assessment by a foetal medicine specialist,^6^ and who met clinical criteria as described by the International Federation of Obstetrics and Gynaecology (FIGO) classification^2^ at the time of laparotomy, with final histopathological confirmation of PAS as examined by a perinatal histopathologist (>10 years of experience) for cases of Caesarean hysterectomy or myometrial resection (Figure 1A and B). Placenta previa was defined as the placenta covering the internal cervical os, while a low lying placenta was defined as when the leading edge of the placenta was within 20 mm of the internal cervical os.^8^ All participants were cared for by a PAS multi-disciplinary team.^24^

For centre 3, data were obtained from a collaboration site with an established PAS MDT service.^25^^,^^26^ Data were obtained retrospectively from studies between January 2019 and January 2023. Inclusion criteria were as for centre 1, with women who had an MRI for suspicion of PAS from ultrasound findings, which was confirmed either clinically and/or histopathologically as per the FIGO criteria were eligible to participate.^2^

The Radiomics Quality Score (RQS)^16^ and CheckList for EvaluAtion of Radiomics research (CLEAR),^27^ which are necessary for the transparent reporting of radiomics studies, are included in Supplementary Material.

### MR image acquisition

In centres 1 and 2, patients were scanned on a GE Optima 450 W 1.5 T MRI scanner (GE Healthcare, Waukesha, United States) (*N* = 36) or a 1.5 T MAGNETOM Sola (Siemens Healthineers, Erlangen, Germany) (*N* = 8), while in centre 3 all patients (*n* = 56) were scanned on a HDxT GE 3 T (GE Healthcare, Waukesha, United States). For all centres, scans were performed using a T2-weighted sagittal 2D balanced steady-state free precession (b-SSFP) (FIESTA sequence for GE system MRI and TrueFISP for Siemens) acquisition sequence with a slice thickness of 3-4 mm and slice spacing of 1 mm.

### Radiomics processing

**Figure 2.**
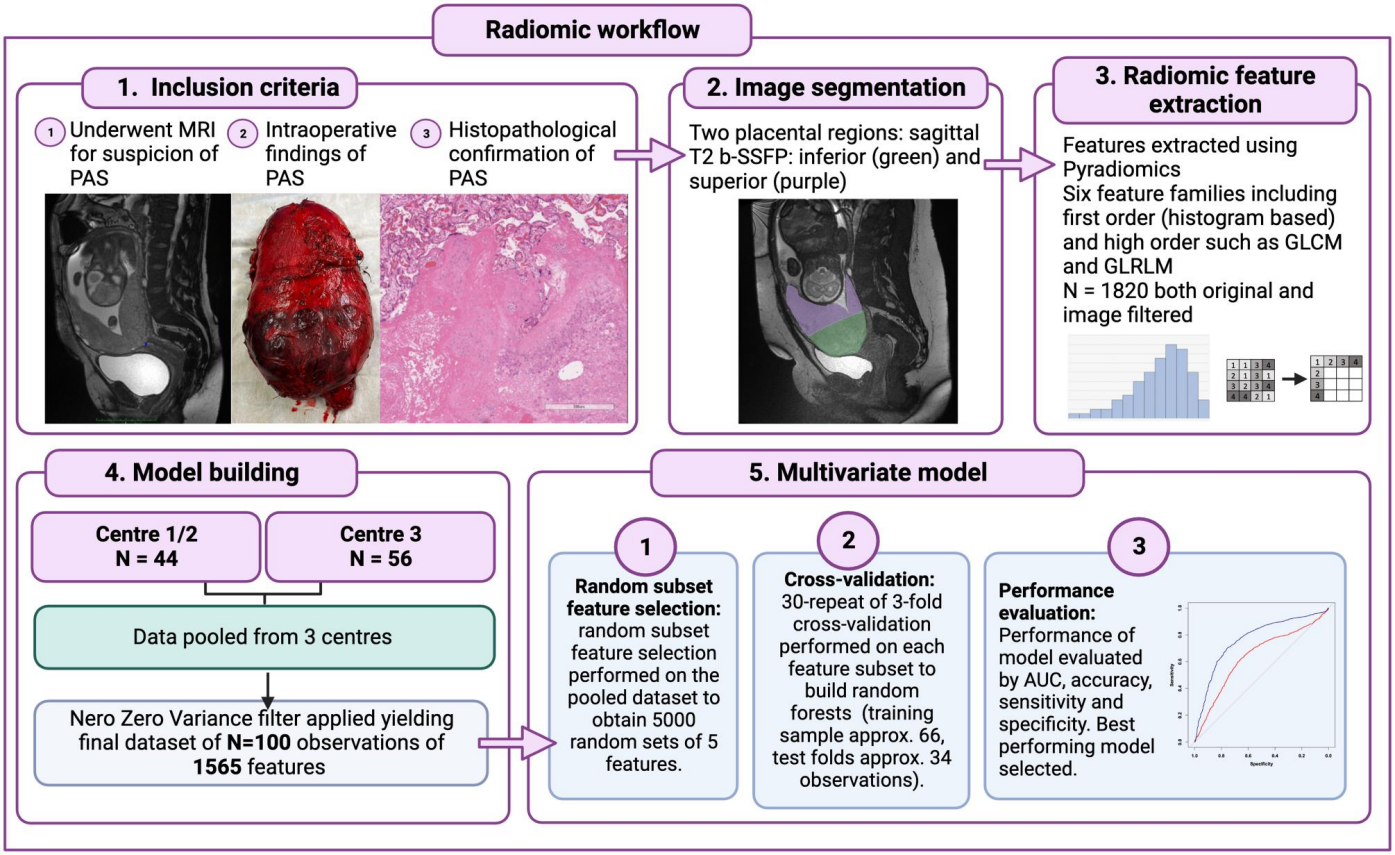
Steps in radiomic processing. A summary of the radiomic workflow.

The workflow for radiomics processing is summarized in Figure 2. The code for these steps and methodology used is publicly available in the following repository (R code).

### Image segmentation

Our group previously published detailed methodology of the placental segmentation protocol.^22^ In brief, for the purpose of radiomic feature extraction, 2 placental regions of interest (ROIs) were manually segmented onto multiple representative slices in the sagittal plane using Image processing software OsiriX (open-source software; www.osirixviewer.com); an inferior placental ROI and a superior placental ROI (Figure 1C and D). Segmentations were performed by 3 independent investigators (HB 5 years experience, DB specialist consultant radiologist >20 years of experience, RM specialist registrar radiologist 7 years of experience) who were blinded to the final clinical/histopathological outcome.

### Feature extraction

Image processing and radiomic feature extraction were performed using PyRadiomics.^16^ Radiomic features extracted from Pyradiomics have previously been mathematically defined and described, with definitions available here.^28^ Gray value normalization was performed using a scale of 100, a fixed bin width of 32 was used for image discretization and the voxel array shift was set to 300.^29^ Feature extraction resulted in the following features being obtained: 106 radiomic features from 6 feature families including shape, first-order (histogram-based) and second-order [Gray Level Cooccurrence Matrix (GLCM), Gray Level Run Length Matrix (GLRLM), Gray Level Size Zone Matrix (GLSZM), and Gray Level Dependence Matrix (GLDM)) features], from the original image. In addition, convolutional image filters were applied including Laplacian of Gaussian (Log) (LoG with 5 sigma levels, 1 level of wavelet decompositions resulting in 8 derived images and images derived using square, square root, logarithm, and exponential filters) resulting in another 1714 (total 1820) radiomic features being extracted.^21^

### Preliminary exploration of the radiomic feature set

A near-zero-variance filter was applied to the subset of radiomic features. Initially, consensus clustering of radiomic features within the pooled dataset (*n* = 100) was performed on the basis of hierarchical clustering of the Euclidean distances between features, to explore how radiomic features may naturally associate with PAS grade. The output was visualized using a heatmap, and scaled principle component analysis (PCA) was also performed on the whole radiomic feature set to assess the distribution PAS grade cases with respect to the first 2 principle components.

### Predictive modelling

Repeated 3-fold cross-validation using 30 repetitions was used on the combined dataset of 100 observations, to assess average model performance on a total of 90 test resamples. This conventional framework reduces bias in evaluation of model generalization. Training subsets therefore contained either 65 or 66 observations, and test folds either 35 or 34 observations. Predictive performance of the univariate and multivariate models considered was assessed on the basis of the area under the receiver operating characteristic curve (AUC) analysis and of overall prediction accuracy. Univariate logistic regression models were trained and tested using the repeated 3-fold cross-validation framework described above to identify inherent predictive potential among the radiomic feature set. The effect of any feature was deemed significant if the *P*-value associated with its model fit was below 5% after correction for false discovery rate (FDR). Radiomic features and 2 clinical variables (maternal age and number of previous caesarean sections) were included for predictive modelling. Since the cohort size (relative to the number of features available) prohibits effective feature selection for optimal predictive modelling, the potential of radiomics for multivariate prediction of PAS was assessed based on a suboptimal, randomized selection approach.^30^ A total of 5000 random sets of 5 features (out of a total of over 7.7 × 10^13^ such combinations) were taken out of the 1565 available features to train as many random forest (RF) classifiers. Constraining the model size to 5 features meant that a ratio of about 13 observations per feature (since the models were trained on about 66 observations in the cross-validation framework) was available for model training. These 5000 models were evaluated with respect to their cross-validated AUC, accuracy, sensitivity, and specificity. As such, this benchmarking approach can be seen as a randomized truncation of an exhaustive best model selection,^31^^,^^32^ which consists in comparing all possible models in terms of their cross-validated prediction performance, but would be numerically impossible with such a large feature set.^32^ The model with highest sensitivity out of these 5000 models are summarized below. To evaluate spuriousness of the results from this modelling pipeline, this process of benchmarking 5000 random 5-feature models was replicated 4 times, thus selecting another 20 000 random models for evaluation, which yielded comparable levels of performance. Finally, a multivariate logistic regression model was trained on the same dataset as a reference method to compare against the random subset feature selection model.

### Statistical software

Statistical analysis for this study was performed in RStudio (version 4.2.2^33^). Comparisons between the centre-specific datasets were carried out on the basis of a 2-sided, 2-sample Mann-Whitney or Chi-square test, as appropriate for non-normal and normally distributed data, and for inferior and superior regions respectively. A difference between distributions was deemed significant when the associated *P*-value was lower than 0.05 after correction for FDR.

## Results

The study included 100 participants (centre 1 *n* = 36, centre 2 *n* = 8, centre 3 *n* = 56). Of these, 36 subjects had clinically and/or histopathologically defined placenta accreta and 64 had placenta increta. All participants had at least one prior caesarean section, and 58 had a caesarean hysterectomy (Table 1).

**Table 1.** Participant demographics.

	*N* = 100
Maternal age (years, mean)	34.6 (3.9)
Parity	2 (2-4)
Number of previous CS	2 (1-2.7)
Gestational age of MRI (weeks)	29 (27-33)^a^
Gestational age at delivery (weeks)	34 (32-36)
Placental location	
Placenta previa	88
Low-lying placenta	10
Other	2
Surgical management (*n*)	
Caesarean hysterectomy	58
Uterine conservation	42
Estimated blood loss (mL)	1000 (561-1687)
PAS grading	
Placenta accreta	36
Placenta increta	64

Data in median (IQR) unless otherwise specified.

a Missing data for *n* = 2.

### Validation of previously described model

Previously, we trained a model using bootstrapping from a smaller sample size including only data from centres 1 and 2 (*n* = 41).^22^ As a first step, we validated the previous model using the new data obtained from centre 3. The results from the validation of the previous model on the new dataset are presented in Supplementary Material, with a support vector machine model achieving the best performance on multivariate analysis with a sensitivity of 0.620 (95% CI: 0.068; 1.0), specificity of 0.619 (95% CI: 0.059; 1.0), an AUC of 0.671 (95% CI: 0.440; 0.922), and an accuracy of 0.602 (95% CI: 0.353; 0.817). This demonstrates that we successfully validated the previous model, however performance metrics remained moderate. Therefore, as described above, we pooled the datasets and developed a new approach for multivariate predictive feature selection.

### Hierarchical clustering of pooled feature set and univariate tests of predictive performance

**Figure 3.**
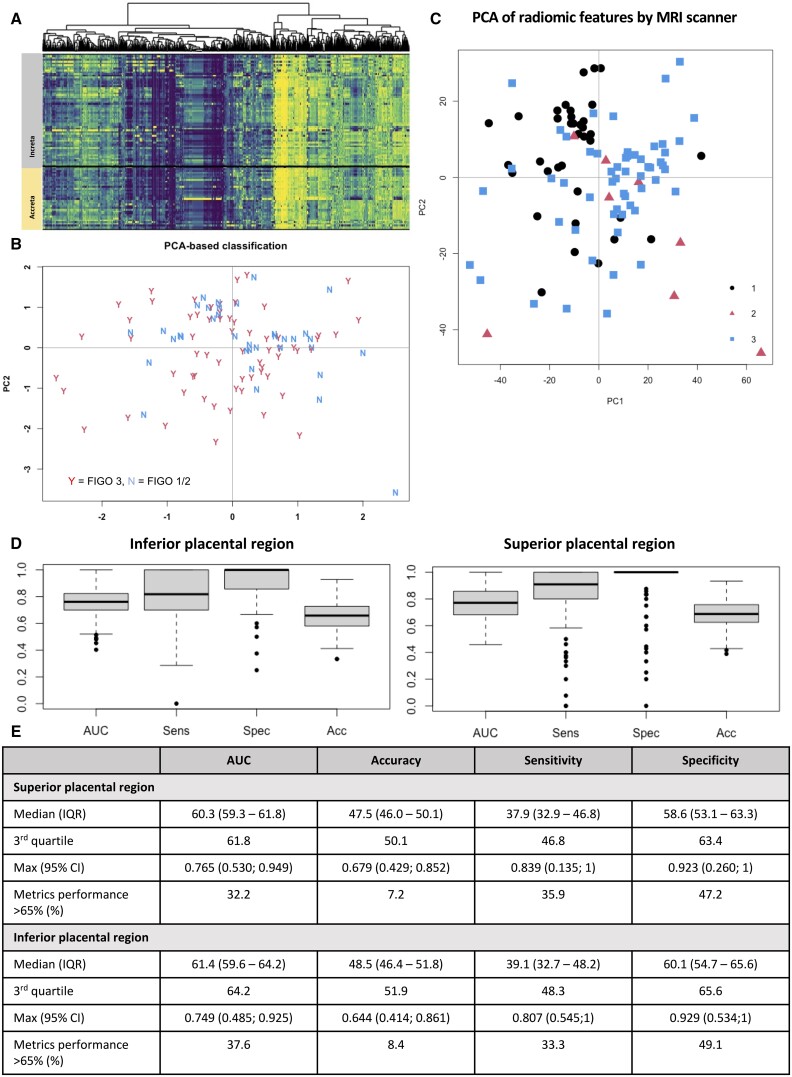
(A) Heatmap showing association of PAS grade and radiomic features. (B) Scaled PCA of radiomic feature set, assessing distribution of PAS grade cases with respect to the first 2 principle components, “Y” indicates placenta increta cases. (C) Scaled PCA of radiomic features by MRI scanner. (D and E) The overall results of the univariate analysis for the inferior and superior placental region, the boxplots in (D) show the performance metrics of the best-performing radiomic features and (E) summarizes the results from the univariate analysis, including the percentage of radiomic features with a performance metric greater than 65%. PAS = placenta accreta spectrum, PCA = principle component analysis, AUC = area under the curve. Sens = sensitivity, Spec = specificity, Acc = accuracy.

Hierarchical clustering, visualized using a heatmap, showed mild structural sub-clusters within each PAS grade group, suggesting that different radiomic phenotypes could be found for each of these groups (Figure 3A). Scaled PCA with respect to the first 2 principle components captured 14.6% of the information (Figure 3B). Some level of separation was visible between mild and severe cases. These exploratory analyses suggested that the radiomic feature set holds potential in separating these cases, but also that this information is hindered by a high feature-to-observation ratio. PCA was performed to assess for separation of radiomic features by scanner type, which did not indicate any evident clustering of features by MRI scanner (Figure 3C). Output from 2-sample, 2-tailed Mann-Whitney tests between any 2 scanners for each feature indicated that 25% of the marginal radiomic feature distributions differed significantly between scanners, after correction of *P*-values for FDR.

On univariate analysis, 37% and 32% of radiomic features achieved an AUC above 0.65, respectively, from the inferior and superior placental ROIs. The results of the univariate analysis (Figure 3D and E) confirm the potential of radiomic features to predict severe PAS cases. These findings, along with the results from the PCA, motivated further multivariate analyses.

### Performance of random-feature selection model

**Figure 4.**
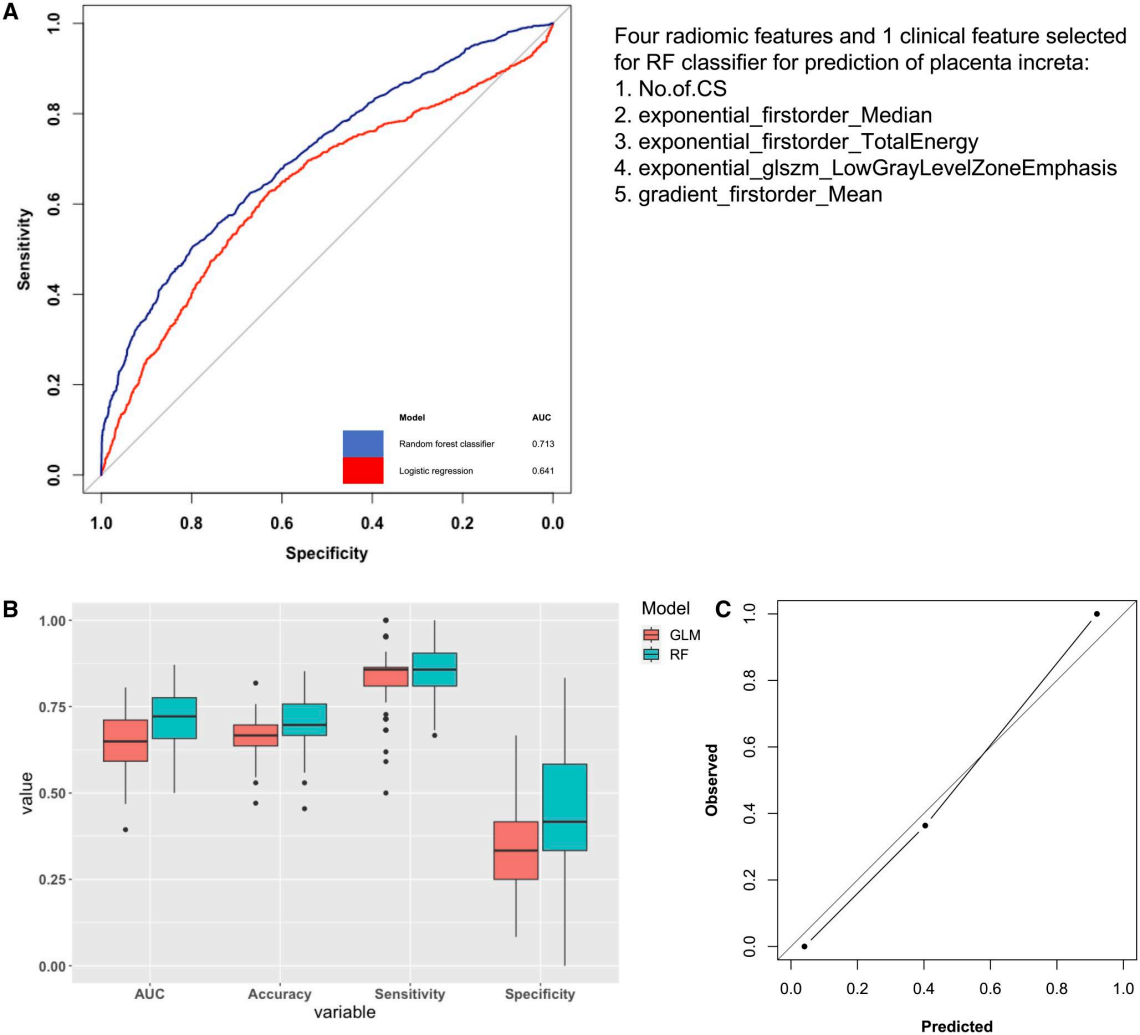
(A and B) The performance of the 5-feature random forest classifier selected from the random subset feature selection scheme. The figures show a comparison of these performance metrics with those obtained from a traditional logistic regression model, to illustrate the benefit of using a typical machine learning modelling approach in order to leverage the potential of radiomic features effectively. (A) ROC curves of the fitted random forest (blue) and logistic regression (red) models for placenta increta classification. (B) Distributions of cross-validated performance metrics of the 5-feature random forest placenta increta classifier obtained *via* random subset feature selection (in blue), and the same distributions obtained by using a conventional logistic regression model with the same features (in red). (C) The calibration curve for the multivariate model for predicted and observed placenta increta cases. The curve indicates there was good agreement between model prediction and observed rates for both placenta increta and placenta accreta cases. PAS = placenta accreta spectrum, GLM = generalized linear model, RF = random forest.

The best 5-feature subset selected *via* the random subset feature selection scheme comprised of 3 first-order variables, one higher-order variable, and a clinical variable, namely exponential_firstorder_Median, exponential_firstorder_TotalEnergy, gradient_firstorder_Mean, exponential_glszm_LowGrayLevelZoneEmphasis, and the number of previous Caesarean sections. The results of that model are shown in Table 2, which achieved an AUC of 0.713 (95% CI: 0.551; 0.854), an accuracy of 0.695 (95% CI: 0.563; 0.793), sensitivity of 0.843 (95% CI: 0.682; 0.990), and specificity of 0.447 (95% CI: 0.167; 0.667). None of the features selected in the final model had significant differences across scanners. Overall, 24% of models trained using random-feature selection yielded an AUC of 70% or higher, and 95% had a sensitivity of 70% or higher. Furthermore, we compared the performance metrics obtained from the random forests model with those obtained from a traditional logistic regression model (Figure 4A and B). We found improved performance metrics by using a typical machine learning modelling approach (RF) in order to leverage the potential of radiomic features effectively.

**Table 2.** Performance metrics of multivariate clinical-radiomic model [mean (95% CI)].

Performance metrics	Clinical-radiomic model superior
AUC	0.713 (0.551; 0.854)
Accuracy	0.695 (0.563; 0.793)
Sensitivity	0.843 (0.682; 0.990)
Specificity	0.447 (0.167; 0.667)

Calibration curve for the multivariate model (Figure 4C) demonstrated good agreement between the predicted and observed rates of severe placenta increta. This suggests that the model was able to estimate the probability of PAS appropriately both for placenta accreta and placenta increta cases.

## Discussion

This study validates our previous multivariate model for classifying severe placenta increta cases and presents a new model with improved performance metrics. We found a subset of randomly selected radiomic features able to identify severe placenta increta cases from T2-weighted MR imaging using a well-defined PAS cohort from a multi-centre dataset. The model demonstrated good sensitivity for using radiomic features to identify placenta increta cases, suggesting there is predictive potential of radiomic features for placenta increta characterization.

A number of studies have applied radiomics to PAS MRI previously.^17–19^ Similar to our approach, others also employed manual segmentations to create ROIs on the placenta.^17^^,^^18^^,^^20^^,^^34^ Combining deep learning and radiomics in a clinical-radiomic model has been explored in a few studies to date with good performance metrics for predicting diagnosis of PAS.^17^^,^^34^ Other PAS studies have implemented radiomics to predict clinical outcomes, such as undergoing caesarean hysterectomy or estimated blood loss.^18^^,^^19^^,^^35^

In PAS radiomics, several studies have shown high performance metrics for predicting the diagnosis of PAS^36–38^ or predicting clinical outcomes such as caesarean hysterectomy^18^^,^^35^ or haemorrhage.^39^^,^^40^ However, predicting the binary presence or absence of PAS is less clinically useful as the condition presents a large spectrum of disease.^2^ Furthermore, prediction of clinical outcomes such as hysterectomy is not very generalizable to other centres as this will depend largely on local practice for the management of PAS, with some centres performing largely uterine conservation or conservative management.^13^^,^^41^ Therefore in this study, we aimed to predict a well-defined clinical and histopathological subtype at the severe end of the spectrum, as it is of greatest clinical benefit to identify these cases which require specialist tertiary care.^4^^,^^42^ This study suggests that the addition of radiomics to antenatal imaging has good sensitivity for identifying these severe cases. This is also more generalizable to other centres, as the identification of these severe cases can then be managed as per local practice.

Furthermore, we have shown that combining datasets with different MRI scanners in order to increase the number of observations is feasible in PAS. It is well described that differences in MRI scanning protocols, sequences, and B strengths, for example, result in different characteristics in the image.^43^^,^^44^ This in turn may influence the radiomic features extracted from different MRI scanners.^45^^,^^46^ To minimize these differences, standard image pre-processing in the form of normalization was performed prior to feature extraction.^47^^,^^48^ Furthermore, PCA of our dataset showed minimal clustering of radiomic features by MRI scanner type (Figure 3C), with no features included in the final model significantly different between MRI scanners. As PAS is a rare condition, any future study with sufficiently large numbers will likely require collaboration between multiple centres and therefore multiple MRI scanners. Here, we have shown this approach is feasible. This is an important step for future radiomic studies as PAS is a rare disease, hence it is challenging to obtain large datasets of a well-defined cohort of cases from single centres.

This study has several strengths and limitations. This study validates our previously developed model to predict severe placenta increta cases, further supporting the feasibility of radiomics in this area. Furthermore, we present a cohort of PAS cases defined using well described ultrasound features, and clinical and/or histopathological grading.^2^^,^^6^ This is an important strength as the PAS literature is significantly limited by the heterogeneity in the diagnosing and reporting of PAS.^21^^,^^49^^,^^50^ Data were obtained from multiple centres and reflect the real clinical scenarios of PAS in 2 different countries, meaning the results are more generalizable. Although segmentations were performed manually, we adherend to a clear annotation protocol and previously found a high ICC between different readers creating these segmentations.^22^ Furthermore, we provide open-source code of our methodology. The RQS^16^ and CLEAR^27^ for this study are included in Supplementary Material, which are 2 initiatives which aim to improve the reporting of radiomics studies, ensuring reproducibility and transparency. The RQS of this study is 47%, which is improved upon previous PAS radiomic studies.^21^

This study is limited by the multi-centre approach which resulted in multiple MRI scanners being used which, as discussed above, will inherently affect the radiomic features extracted. However, despite this we were able to validate our previous model and by pooling the datasets present a model with good performance metrics showing that radiomics for prediction of severe PAS subtypes is feasible. Furthermore, the model was obtained from random exploration, hence not a fully replicable approach. However, this concern may be alleviated by the fact that several randomly discovered models yielded similar levels of performance. There is a risk of overfitting and selection bias with the approach used for feature subset (and therefore the overall model) selection. However, this risk may be considered acceptable here as an exhaustive search, in which every model would be trained and tested, and the best-performing model (in terms of test-set AUC) would be chosen as final model, is not realistic for this dataset as it would consist in training over 7.7 × 10^13^ 5-feature models. The approach taken here consists instead in considering a randomized subset of models in a best-subset-selection-like approach. Some degree of overfitting is unavoidable in such frameworks, but again may be considered reasonable as the aim of this study was to build up a body of evidence for the potential of a typical radiomics approach for prediction of PAS. Note the model is no longer tweaked once it is chosen, which minimizes the risk of introducing further bias. Furthermore, while we performed manual segmentations of the placenta, other ROIs such as of the retroplacental myometrium may provide additional performance metrics for the prediction of PAS.^51^ Finally, radiomics is an emerging field in medicine and therefore still pending of standardization in many areas, including harmonization in multi-centre studies.^52–54^

In summary, we validate our previously published model and present a new radiomics based approach for predicting severe placenta increta cases. This study, using a well-defined PAS cohort, demonstrates the feasibility of using radiomics to identify severe clinical and histopathological PAS subtypes. This is of clinical importance as PAS is a high-risk complication of pregnancy with a rising incidence. Therefore, predicting disease severity antenatally is important to identify cases which need specialist tertiary care. We have shown that radiomic features have potential to identify severe cases.

## Supplementary Material

tqae164_Supplementary_Data
