# Supplementary material for: Radiomic study of antenatal prediction of severe placenta accreta spectrum from MRI
Source: Br J Radiol. 2024 Aug 17;97(1163):1833–42. doi: 10.1093/bjr/tqae164 (PMC11491593; doi:10.1093/bjr/tqae164)
Supplement: tqae164_Supplementary_Data [file tqae164_supplementary_data.zip › tqae164_Supplementary_Data/Supplementary materials Table 1.docx]

**Table 1: Supplementary materials**

| **Performance metrics (95% CI)** | **Test** | **External validation** |
| --- | --- | --- |
| AUC | 0.64 (0.29; 0.93) | 0.67 (0.44; 0.92) |
| Accuracy | 0.61 (0.38; 0.84) | 0.60 (0.35; 0.82) |
| Sensitivity | 0.68 (0.31; 1.0) | 0.62 (0.07; 1.0) |
| Specificity | 0.49 (0.20; 1.0) | 0.62 (0.06; 1.0) |

Table shows the results from the original multivariate bootstrapped model using a Support Vector Machine (SVM), and the results from validation on the new dataset. Prior to validation, the SVM was retrained without the clinical variable Body Mass Index (BMI), as this was not available in the new dataset. Of note, this variable was not included in final feature selection for model training. However, performance metrics remained moderate in the external validation, and therefore the datasets were pooled and a new approach for multivariate predictive feature selection was developed.
